# Evaluation of RuralkidsGPS; A Novel Integrated Paediatric Care Coordination Model of Care in Rural Australia – a Mixed-Methods Study Protocol

**DOI:** 10.5334/ijic.7008

**Published:** 2023-11-24

**Authors:** Raghu Lingam, Hayley Smithers-Sheedy, Stephanie Hodgson, Karen Hutchinson, Tammy Meyers Morris, Nan Hu, Natasha Nassar, Elizabeth-Ann Schroeder, Rezwanul Rana, Emma Dickins, Kirsten Bula, Yvonne Zurynski

**Affiliations:** 1 Population Child Health Research Group, School of Women’s and Children’s Health, University of New South Wales, Sydney, NSW, Australia; 2 The Sydney Children’s Hospitals Network, Sydney, NSW, Australia; 3 Australian Institute of Health Innovation, Faculty of Medicine, Health and Human Sciences, Macquarie University, Sydney, NSW, Australia; 4 Department of Community Paediatrics, Sydney Children’s Hospital, Randwick, NSW, Australia; 5 Child Population and Translational Health Research, Children’s Hospital at Westmead Clinical School, Faculty of Medicine and Health, The University of Sydney, Sydney, NSW, Australia; 6 Macquarie University Centre for the Health Economy, Australian Institute of Health Innovation, Macquarie University, Sydney NSW 2113, Australia

**Keywords:** model of care, care coordination, rural, children and young people, implementation, medical complexity

## Abstract

**Introduction:**

The Kids Guided Personalised Service (KidsGPS) is an integrated model of care coordination for children and young people (CYP) living with medical complexity. After successful implementation in an urban setting, the model of care will be rolled-out at scale to four rural regions in New South Wales, Australia to establish RuralKidsGPS. This paper describes the approach and methods for the outcome and implementation evaluation of RuralKidsGPS.

**Description:**

The evaluation aims to assess health, economic and implementation outcomes and processes whilst identifying barriers and enablers to inform future rollouts. Measures of health service utilisation (primary outcome), child health related quality of life and parent/carer experiences will be assessed. The implementation evaluation will occur alongside the outcomes evaluation and is underpinned by the Consolidated Framework for Implementation Research and informed by validated quantitative measures and qualitative interviews with patients, families, healthcare providers and service managers. An economic analysis will determine incremental cost effectiveness ratios for the new model of care using health service utilisation data.

**Conclusion:**

RuralKidsGPS, if effective, has the potential to improve equity of access to integrated care for CYP and their families and this protocol may inform other evaluations of similar models of care delivered at scale.

## Introduction

Children and young people (CYP) who are medically complex have multiple and/or significant chronic health problems. This group represents approximately 10% of patients attending children’s hospitals, but accounts for 60% of expenditure [1,2]. CYP who are medically complex require support from different health care teams frequently based across more than one service and location. In the absence of planned coordinated care for these CYP, missed and/or duplicated care increases costs for the health system and out-of-pocket expenses for families [3]. The cost of uncoordinated care is estimated to be up to 35% higher than coordinated care [4].

A care coordination service for CYP with chronic and/or complex health conditions, developed at the Sydney Children’s Hospitals Network (SCHN) in Sydney, New South Wales (NSW), Australia, known as Kids Guided Personalised Service (KidsGPS) has already demonstrated successful outcomes in the metropolitan setting [3]. The service is focussed on CYP with increased care coordination needs due to several factors, including medical complexity, vulnerability, involvement of multiple health care teams, and reduced access to appropriate services. Evaluation of KidsGPS demonstrated a 40% reduction in emergency department (ED) presentations and 42% reduction in day-only admissions over 2 years for 534 CYP who had over 50 different diagnoses [3]. Through the adoption of KidsGPS, approximately $2.5 million/annum was saved for the tertiary hospital network, and >50,000 km of family travel was prevented [3]. Systematic review and meta-analyses data published by our research team have indicated that paediatric integrated care interventions significantly improve CYP related quality of life scores compared to usual care [5].

In NSW, Australia, 500,000 children, 30% of the state’s total, live outside major cities. To address the healthcare needs of this population, Rural Kids Guided Personalised Service (RuralKidsGPS) was co-designed with parents and professionals as an innovative child-centred model of care for CYP living with medical complexity in rural and remote regions. Within the RuralKidsGPS model, a new role was established in each LHD for a paediatric care coordinator responsible for facilitating care integration and establishing a circle of coordination formed by the leads from the health service, community and family [1]. The paediatric care coordinators work closely with local and tertiary care teams at SCHN, and families, to meet the CYP’s needs through the development of shared care plans and technology-enabled healthcare. Importantly, the RuralKidsGPS model of care aims to build capacity for the family and local healthcare teams.

This paper describes a research protocol aimed at evaluating the impact of RuralKidsGPS on health service utilisation, implementation outcomes, barriers and enablers to delivering RuralKidsGPS and the cost-effectiveness of this program for families and the healthcare system.

## Methodology

### Study design

**Figure 1:**
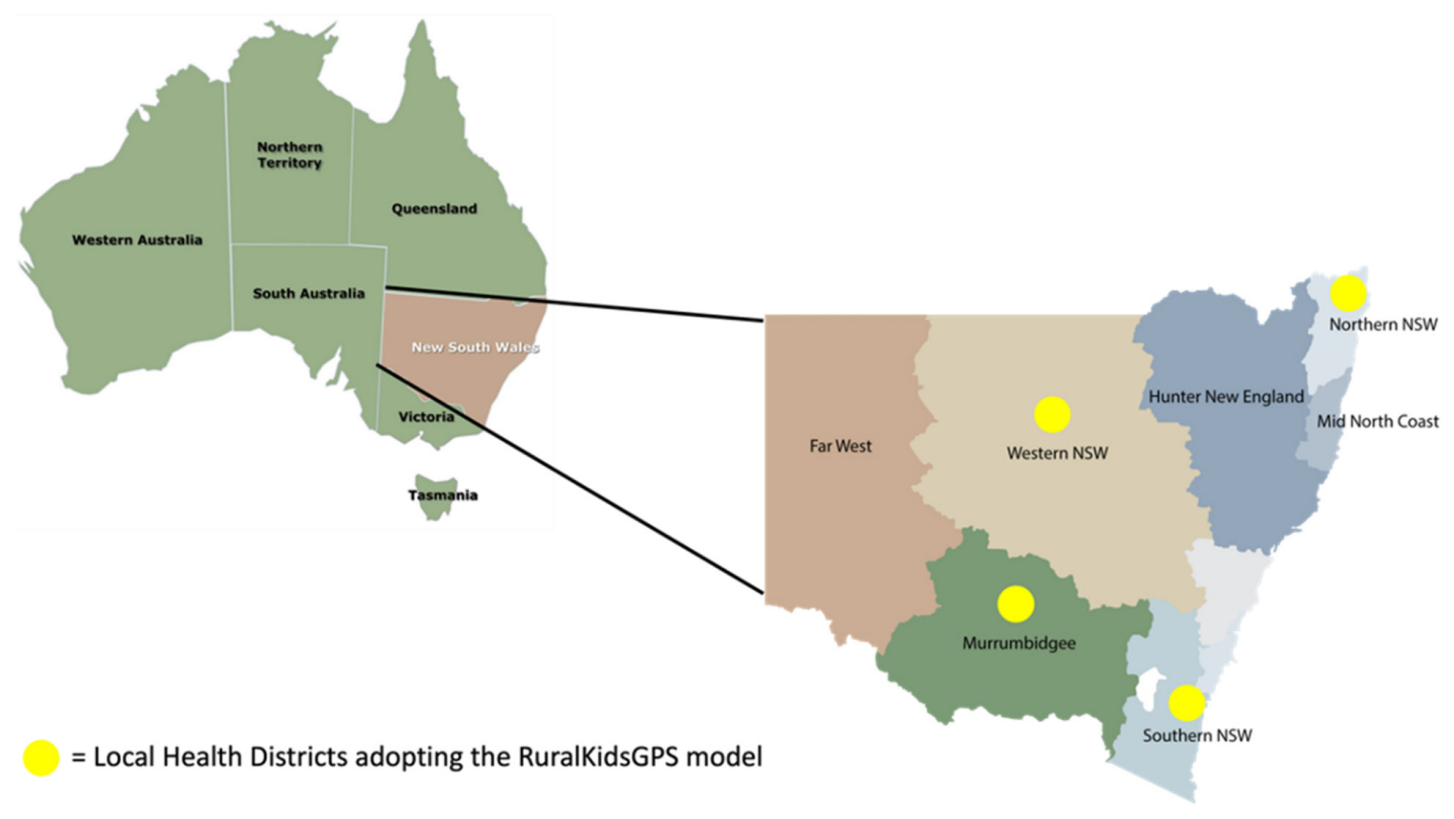
Local Health Districts involved in the RuralKidsGPS study.

The evaluation of RuralKidsGPS will consist of an integrated impact, implementation and economic evaluation embedded within a quasi-experimental study across four rural NSW local health districts (LHDs) in Southern, Northern and Western New South Wales and Murrumbidgee (Figure 1).

The primary aim of this evaluation is to assess the impact of RuralKidsGPS on health service utilisation (ED presentations, hospital admissions, and outpatient (OP) appointments), among CYP enrolled in RuralKidsGPS intervention compared to usual care.

The secondary aims are to:

- Describe trends in health service utilisation (ED presentations, hospital admissions, OP appointments, and general practice (GP) attendances) over time;
- Assess the change in parent reported satisfaction with care, parental wellbeing, and parent and CYP reported quality of life at enrolment, at 6 and 12 months after recruitment to RuralKidsGPS;
- Assess the implementation of RuralKidsGPS model from the perspective of families, CYP, healthcare providers, managers, and practitioners, and factors that help or hinder this implementation to inform further adaption of the model and ensure scalability;
- Assess the cost-effectiveness of RuralKidsGPS model of care compared with standard care.

### Ethics

This study will be guided by the RuralKidsGPS Steering and Evidence Translation Committee which includes in its membership representatives from all participating local health districts and consumers. Ethical oversite for this study is held by the SCHN Human Research Ethics Committee (2021/ETH01055).

### Study cohort

The study population includes CYP with medical complexity aged 0 to 18 years, referred for care coordination, who meet the following criteria:

– A diagnosis expected to last at least 12 months
– And if ≥ 6months of age, frequent use of health services within the last six-month period, defined as:
  - Two or more ED presentations, OR
  - Hospital admission for seven days or more, OR
  - Two or more outpatient appointments.

A cohort of CYP meeting these criteria and referred for care coordination, their parents and guardians, and clinical and non-clinical staff involved in the planning and/or delivery of the RuralKidsGPS model of care, will all form a part of the study cohort.

### Recruitment

Children meeting the inclusion criteria for care coordination as described above will be recruited within each of the four LHDs. For children enrolled into the local service, the paediatric care coordinator (PCC) will discuss with each family their potential participation in the evaluation project. After providing a brief overview of the project, the PCC will ask permission for the contact details (parent name, contact number, email, and child name) to be shared with the researcher embedded with the local team. The embedded researcher will then contact the parent/carer and provide them with the participant information sheet and consent form.

Clinical staff (e.g. healthcare providers), and non-clinical staff (e.g. service managers) involved in the care coordination of the children will be recruited from each of the four LHDs and SCHN. Members of the research team will make direct contact with clinical and non-clinical staff and will provide them with an information sheet and consent form which describes what is involved in the research and that participation is voluntary. Upon agreement and consent, the implementation evaluation team will organise a suitable time to conduct the interview or focus group. Links to study surveys will be sent via an invitation email including the participant information form. These forms will also will be embedded in the electronic surveys and participants will be asked to confirm that they have read these and that they consent prior to completing the survey.

### Intervention

**Figure 2:**
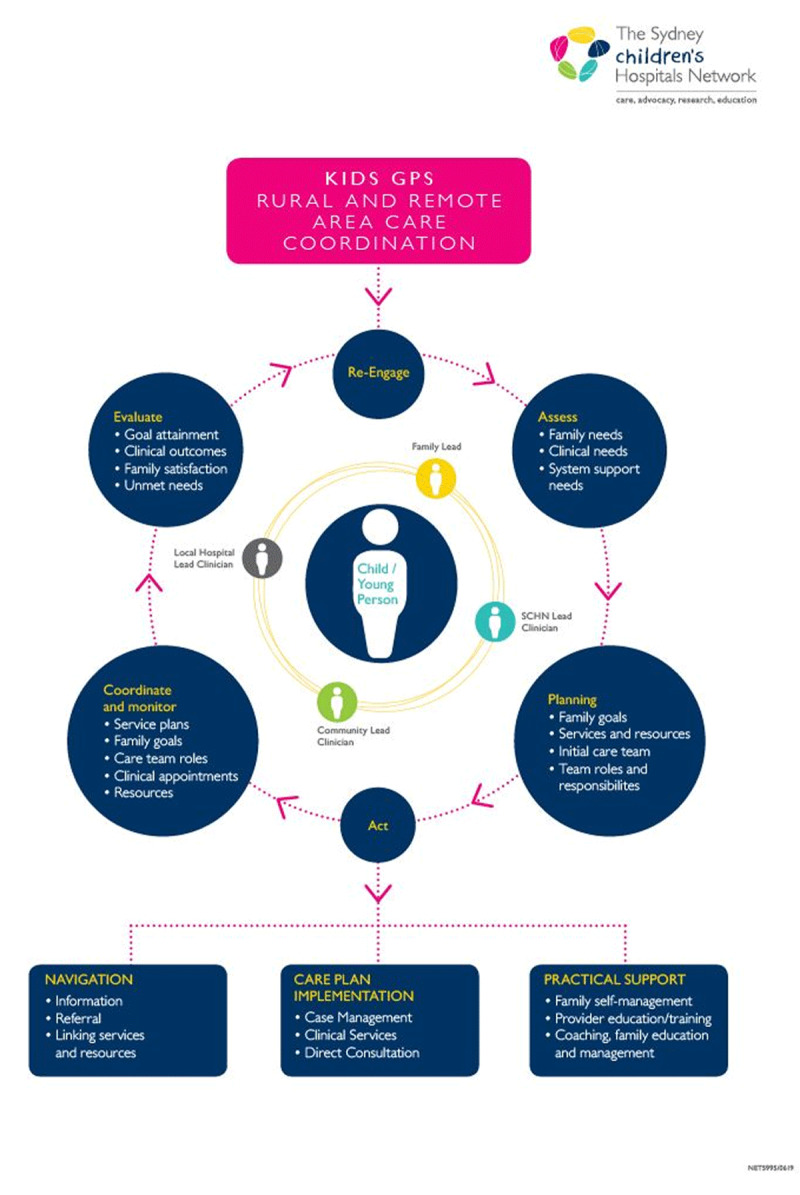
RuralKidsGPS Circle of Coordination (adapted from Cohen, et al. 2011).

The RuralKidsGPS model uses the circle of coordination for CYP with medical complexity, which places the CYP and their needs at the centre of the system for integrating care (Figure 2). Families are guided by local PCCs who are responsible for facilitating care integration and establishing the circle of coordination which is formed by leads from the hospital, community, and family.

The PCCs work with families to:

- Understand their personalised needs.
- Develop and implement an integrated family-centred shared care plan.
- Build capacity for family self-management and deliver practical holistic support.

The PCCs also work with treating teams to identify opportunities to integrate and share care among central units and local teams, deliver virtually enabled care, streamline appointments, avoid unnecessary hospitalisations, and prevent avoidable ED attendances.

### Impact evaluation

#### Health Service Use

**Figure 3:**
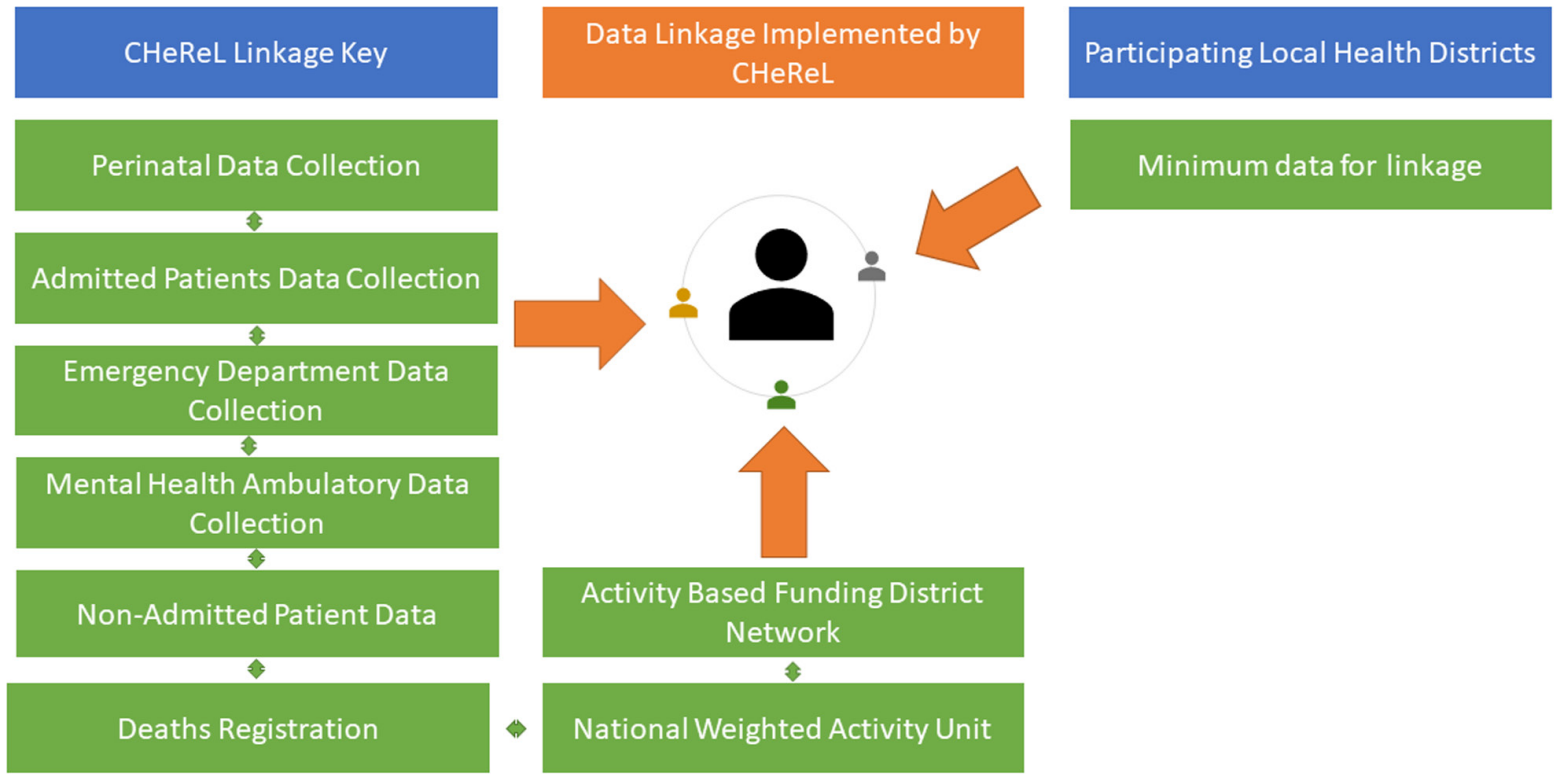
Sources of data for linkage.

A cohort-level evaluation of health utilisation (primary outcome) will be conducted. A cohort of children receiving the RuralKidsGPS will be recruited from the participating LHDs. Routinely collected administrative health data for these children will be requested and linked by an independent group, the NSW Centre for Health Record Linkage (CHeReL) (Figure 3). Using this deidentified linked data, we will compare the change and trend in health utilisation of children (ED presentations, hospital admissions, and outpatient (OP) appointments) who received RuralKidsGPS, to that of a matched comparison group.

The comparison group will be identified using the linked administrative health data (ED presentations, hospital admissions, and OP appointments) and frequency matched with 20 children (to each case) by clinical diagnosis, year of birth, sex, local health district who and meet the eligibility criteria for health service use within the past 6 months (outlined in the Study Cohort section above) but who did not access RuralKidsGPS. A population-level evaluation will also be conducted which will include all CYP in the 4 health districts meeting the inclusion criteria.

#### Impact Evaluation: Patient and family reported outcomes data collection

In our intervention cohort, secondary child and parent reported outcomes will assess family reported experiences and parental satisfaction with care integration (PICS) [6], out of pocket expenses (adapted Mumford Scale) [7], parent and child quality of life (EQ5D5L) [8], (PedsQL4.0) [9], (CHU9D) [10] and parental wellbeing (WEMWBS) [11]. These measures and basic demographic data (e.g. date of birth, postcode for estimation of Indexes of Relative Advantage and Disadvantage [12], gender, Aboriginal and Torres Strait Islander Status) will be collected via phone and secure electronic survey by a researcher embedded in each of the LHDs, at three time points: enrolment, 6 months and 12 months later to enable longitudinal comparisons, (Table 1).

**Table 1:** Family reported outcome measures.


OUTCOME MEASURES	PURPOSE

Paediatric Integrated Care Survey (PICS) [6]	Measures experiences of integration of child’s care

Adapted Mumford scale [7]	Measuring non-medical out of pocket expenses for families

EuroQol 5 Dimension 5 Level (EQ-5D-5L) [8]	Health status measure

Paediatric Quality of Life Measure (PedsQL4.0) [9]	Measuring health paediatric health outcomes

Child Health Utility 9D (CHU 9D) [10]	Instrument used to adjust quality of life years from PedsQL4.0 score for economic evaluation

Warwick-Edinburgh Mental Well-being Scale (WEMWBS) [11]	Understanding and measuring mental health and wellbeing


#### Impact Evaluation Analyses

Based on local data from the participating LHDs, we aim to recruit 400 children across the four LHDs. We will match each case by 20 controls, resulting in a total of 8,000 controls. This will provide 90% power to detect a 20% difference in the number of health service use occasions, including outpatient visits and hospital admissions between intervention and comparison groups. Having 400 cases will also provide the power of 90% to detect a within-group 20% decrease in the number of health service use occasions. This sample size will also allow us to detect a clinically important difference (4.36 points) for child health related quality of life in children post- vs pre-intervention change in the PedsQL4.0 Total Scale Score [9].

We will calculate rate of health service use (e.g. ED, hospital admissions and OP visits) in person-years for the intervention and comparison groups during the baseline (3 years prior to enrolment) and the intervention period (12 months after enrolment), respectively. To assess the effectiveness of this intervention, we will conduct multivariable Poisson (negative binomial if over-dispersion is present) regression analysis with intervention status (intervention versus comparison) as the main independent variable and the rate of health service use (ED, hospital admissions and OP visits, separately and combined) during the intervention period as the outcome variable.

To examine how health service use changes over time between intervention and comparison groups, we will analyse rates of health service use during baseline and intervention periods using mixed effect Poisson regression models, including the interaction term between intervention status and time (after RuralKidsGPS implementation versus before RuralKidsGPS). In all regression analyses, we will control for participant socio-demographics (e.g., age at enrolment, sex, Aboriginal and Torres Strait Islander status, socio-economic status) and clinical factors (e.g., type and severity of health conditions), and baseline rate of health service use. Considering potential clustering effect by site, we will use the generalised estimating equations (GEE) approach in the regression analysis.

**Figure 4:**
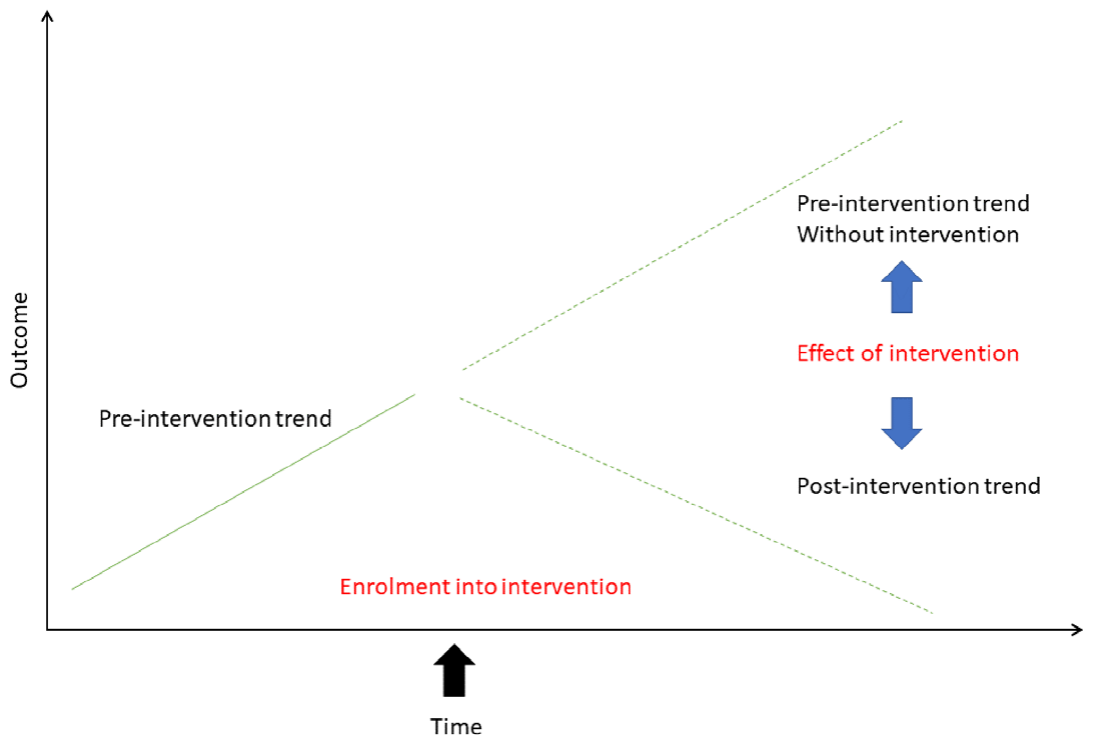
Using interrupted time series analysis to measure change before and after intervention. Adapted from Kontopantelis et al 2015 [13].

Additionally, we will plot monthly rate of health service use for both intervention and comparison groups over baseline and intervention periods. We will undertake an interrupted time series (ITS) analysis to gain a better understanding of how the effect of intervention changes over time (e.g. to identify when the intervention starts to take effect). ITS is one of the most effective quasi-experimental evaluation methods and has been used in many health intervention evaluations [13]. An ITS analysis allows us to control for time-varying confounders such as seasonality and co-occurring competing interventions, thereby addressing important threats to internal validity and causal inference. Figure 4 illustrates using ITS to measure change before and after intervention.

Regarding patient and family reported outcomes, we will describe them at each survey time point using mean and standard deviation, or median and inter-quartile range. We will conduct regression analysis of these outcomes at each survey (pre-intervention, 6-month and 12-month post-intervention), respectively, with intervention status as the main independent variable. We will control for participants’ socio-demographic, clinical and baseline outcome variables, and use the GEE approach to account for potential clustering effect by site. We will conduct intention-to-treat analysis.

### Implementation Evaluation

We will undertake a mixed methods implementation evaluation, underpinned by the Consolidated Framework for Implementation Research (CFIR) [14]. The CFIR is a comprehensive meta-theoretical framework that provides a taxonomy of standardised operationally defined constructs to guide systematic assessment in the identification of factors (barriers and enablers) influencing implementation of complex programs within complex contexts [14]. Contextually relevant strategies for successful implementation as well as barriers and facilitators for adoption, delivery and sustainability will be identified to inform future rollouts of the RuralKidsGPS model of care. The mixed methods research will also assess implementation outcomes including acceptability, appropriateness, and adaptability of the RuralKidsGPS model as viewed by children and families, healthcare providers, and service managers, informing answers to specific questions that reflect implementation outcomes proposed by Proctor et al., (2011) (Table 2) [15].

**Table 2:** Defining implementation outcomes for RuralKidsGPS, based on Proctor’s framework.


OUTCOME	QUESTION

Acceptability	Do clinician and non-clinicians, parents and children view RuralKidsGPS as agreeable?

Adoption	To what extent do clinicians and parents use RuralKidsGPS?

Appropriateness	Do all stakeholders perceive RuralKidsGPS as relevant & useful?

Fidelity	Is RuralKidsGPS applied as intended? Are all component parts of the intervention delivered as planned?

Feasibility	Are the component parts of RuralKidsGPS practical to deliver within the service?

Coverage	How many service users of those eligible are reached?

Cost*	How much does it cost to successfully implement RuralKidsGPS?

Sustainability	What are the factors that will allow RuralKidsGPS to be scaled-up further?


*This outcome will be informed by the economic evaluation.

#### Implementation evaluation qualitative data collection

Semi-structured interviews will be undertaken by trained researchers with CYP aged over seven years, their parents, or caregivers, after they have been accessing RuralKidsGPS for at least 6 months. Children aged over seven years are considered capable of understanding the purpose of the research and of making informed decisions as to whether to participate in a brief interview or not [16].

Healthcare providers and managers involved in delivering the model of care will also be interviewed between 6 and 12 months after implementation of RuralKidsGPS. Purposive sampling will be used to recruit a diverse sample of service users (children, young people, and families) and providers across the four sites. Key stakeholder workshops held within each LHD, will present findings to enable reflection on the appropriateness and acceptability of the model, identify future adjustments to the model and implementation strategies with consideration of local contexts. The outcomes of the workshops will inform the development of an implementation guide to support future scaling up of RuralKidsGPS.

#### Implementation evaluation quantitative data collection

Data will be collected for each child before and after enrolment in the RuralKidsGPS care coordination model. In addition, key LHD staff involved in the delivery or management of RuralKidsGPS will be sent a link to a secure online survey, which includes the Acceptability, Appropriateness and Feasibility measure [15], Normalization Process Theory (NoMAD) tool [17] and Routinisation and Sustainability of work practices in long term care [18], at three time points: enrolment, 6 months and 12 months later as described in Table 3.

**Table 3:** Quantitative data collection for children and families, clinician and non-clinicians at each of the four sites.


GROUP	INSTRUMENT	BASELINE (ENROLMENT)	6 MONTHS	12 MONTHS

Families	Paediatric Integrated Care Survey [6]	x	x	x

Clinician and non-clinicians	Intervention Acceptability, Appropriateness and Feasibility measure [15]	x	x	x

Clinicians and non-clinicians	NoMAD (Normalization Process Theory tool) [17]		x	x

Clinician and non-clinicians	Routinisation and sustainability of work practices in long term care [18]			x


#### Implementation evaluation analyses

All instruments will be scored according to the manuals provided by the authors who initially developed these tools. Measures will be compared over time using t-tests for simple comparisons, or analysis of variance (ANOVA) for comparisons across three time points based on available data (see Table 3). The Routinization and Sustainability of work practices in long term care tool [18] will be used only after RuralKidsGPS has been operational for at least 12 months, providing a one-time measure. This may be repeated in the future if it is decided that the model of care will continue to be supported beyond the current study period.

The three-step iterative process for coding the interview and focus group data will be used to ensure intercoder reliability [19]. The initial stage will involve researchers using the CFIR to independently code an interview transcript. This coding along with inductive insights identified in transcripts will be compared for reliability among the research team and any inconsistencies will be discussed and agreed upon. This process has been shown to lead to a high degree of intercoder reliability among the research team [20]. Once intercoder reliability is established, the remainder of the transcripts will be coded. We will be guided by the Consolidated criteria for Reporting Qualitative research (COREQ) checklist for qualitative research [21]. Qualitative and quantitative data will be synthesised through holistic triangulation [22] to arrive at a deep understanding of the data to answer the questions posed in Table 4.

**Table 4:** Sources, measures, and tools for evaluation.


RESEARCH QUESTION	DATA SOURCE

*1. What is the impact of RuralKidsGPS on health service use?*	Linked data from multiple sources Figure 3

*2. What is the impact of RuralKidsGPS on family reported experiences, and parental satisfaction with care and care coordination?*	Paediatric Integrated Care Survey [6]

*3. What is the family impact related to the child’s healthcare needs (travel and accommodation costs, care for siblings, missed work, missed school days)?*	Adapted Mumford Survey [7].

*4. What is the impact of RuralKidsGPS on parent and child related Quality of Life?*	Parents: EQ-5D-5L [8] Children aged <7 years: Parent reported Paediatric Quality of Life Inventory [9] CYP aged 7–17 years: CHU9D [10]

*5. What is the impact of RuralKidsGPS on Parental mental wellbeing?*	Warwick-Edinburgh Mental Well-being scale [11]

*6. What is the cost of implementing the model of care? What is the cost effectiveness?*	Average costs per encounter in health service use (ED presentation, hospital admission, outpatients encounter); family cost estimates according to adapted method of Mumford et al., 2018 [7]; Quality adjusted life-years (QALYs)

*7. What are the aspects of the model of care and its implementation that support feasibility, acceptability and contextual appropriateness?*	Qualitative semi-structured interviews guided by the Consolidated Framework for Implementation Research [25]; and the Proctor Implementation Outcomes Framework [15].

*8. What are the barriers and enablers for implementing the model at scale?*	Qualitative semi-structured interviews guided by the Consolidated Framework for Implementation Research [25]; and the Proctor Implementation Outcomes Framework [15].

*9. What are the factors that will allow RuralKidsGPS to be scaled-up further?*	Survey for healthcare professionals, researchers and health care managers that includes validated tools: Short intervention acceptability, appropriateness, and feasibility measure [15], Normalization Process Theory Nomad Tool [17], Routinisation and sustainability of work practices in long term care [18].


Using NVivo qualitative data analysis software [23] we will undertake thematic analysis taking a hybrid inductive/deductive approach [24] to identify factors that help or hinder implementation, and these factors will be categorised under the CFIR constructs. Thematic analysis will specifically focus on barriers and enablers of effective behaviour change in terms of engagement with services and operation of the new model of care, whilst taking local site contextual factors into account. Such factors may include details of the setting, resource availability, staff attitudes, capabilities and capacity, and level of support from management.

### Economic evaluation

An economic analysis will determine incremental cost-effectiveness for the new model of care. Health utilisation data will combine the costs of health services pre and post enrolment for each enrolled child. Average costs per activity (ED presentation, hospital admission, OP visit, GP attendance) will be applied to estimate differences in total costs. Resource use will be multiplied with unit costs to calculate the total expenditure per service area and per client before implementation of the new model of care, as well as over the study period and for each of the follow-up intervals. Relevant healthcare utilisation data will be obtained through the data linkage of the routinely collected national administrative datasets. Detailed data will be collected about care coordination, including the type and modality of care provided, duration and personnel involved in care coordination tasks and interactions with enrolled participants from primary sources. The costs to implement the RuralKidsGPS model of care will also be estimated. The study will undertake detailed probabilistic sensitivity analysis and scenario analysis to account for uncertainty within model parameters and model structure.

To estimate broader personal costs incurred by families, we will conduct a parent survey to estimate the costs associated with attending health services for their child, including accommodation, parking, meals, distances travelled (per kilometre average costs for an average vehicle); lost education opportunities (number of school days missed by the child) and missed work time based on estimates published by Mumford et. al. [7]. The cost analysis will be estimated based on the net savings of down-stream resource utilisation and funding impacts of the new model, from a health service perspective (participating health districts, as well as the state’s health system).

Outcome data will capture changes to parent and child reported quality of life assessed using the validated instruments EQ5D5L15 (parent) [8] and Paediatric Quality of Life Inventory (PedsQL4.0), and Child Health Utility 9D (CHU9D) [10] (internal consistency 𝛼 = 0.89 children and 0.92 parent report). These utilities will be included in the estimation of QALYs (quality-adjusted life-years) by calculating the area under the curve, assuming a linear change between the survey dates. QALYs calculated for the year before the intervention starts are based on the health state utility measured at baseline, which was assumed to be stable during the year before.

Cost-effectiveness will be expressed as incremental change in costs (measured by ED presentations, hospital admissions, or OP visits averted), and cost-utility by changes in QALYs gained. Cost-effectiveness will be measured between pre and post implementation of the intervention and calculated according to the formulas below:

\[\begin{array}{l}
Effect\ differences:\ \Delta QALYs = QALY{s_{post\ new\ model\ of\ care}} - QALY{s_{pre\ new\ model\ of\ care}}
\end{array}\]

\[\begin{array}{l}
Cost\ differences:\ \Delta Total\ costs = Total\ cost{s_{postnew\ model\ of\ care}} - Total\ cost{s_{pre\ new\ model\ of\ care}}
\end{array}\]

Imputation methods will be applied to missing values and sensitivity analyses will be conducted for key cost parameters in the data sets to ensure the robustness of the estimates. An additional bootstrap analysis with random 1,000-fold resampling of the original population will be conducted to ensure the validity of confidence intervals.

## Discussion

CYP with medical complexity living in rural and remote areas deserve equitable access to high quality health care, as experienced by their urban counterparts. In Australia, like many other countries, the healthcare system struggles to meet the needs of CYP with medical complexity in general, and particularly those living remotely. This is because our health system is designed for episodic care, provided by health care professionals highly specialised to manage single organ systems, who work in “silos” defined by their medical specialties, institutions, geographical areas, and informal professional networks [26]. Parents/caregivers of CYP with medical complexity and their healthcare providers experience frustration and confusion when accessing and providing healthcare, because of the disconnectedness and complexity of the Australian healthcare system [27].

Paediatric care coordination as a model for CYP with medical complexity, has received more attention in recent years [28,29]. This has been the result of published state and national integrated care strategic frameworks and specific recommendations from paediatric reviews [30,31,32]. While CYP with medical complexity constitute a wide array of different diagnoses, there are similarities amongst this heterogenous group, that lends itself to a diagnoses agnostic model of care, such as RuralKidsGPS [3]. These similar characteristics include; ongoing complex health care needs, frequent usage of multiple health services, need for team-based interdisciplinary care, and for some, use of specialised medical appliances, equipment and disability services [1].

Although the KidsGPS was proven effective in the largest metropolitan children’s hospital network in Australia, its value in rural areas cannot be assumed [3]. The adaptability of the KidsGPS model to rural healthcare settings has not been evaluated, nor has this model undergone rigorous and detailed health economic evaluation to determine whether the costs of the service represent value-for-money through gains in reduced hospital utilization and shifts to lower cost settings (e.g. in primary care) where appropriate. The impacts on quality of life, for children and families, and the views of health professionals and managers implementing these models of care are poorly understood in Australia. These are important considerations for the adaptation and sustainability of such models to varied rural contexts. The mixed methods, integrated impact, implementation, and economic evaluation study outlined in our protocol will provide robust evidence of effectiveness of the model using data from four rural health settings with a geographical coverage of 420,095 km^2^. This evaluation will be the first in Australia to assess the impact, implementation, and cost effectiveness of a novel model of paediatric care coordination that could improve the health and wellbeing of children and families living in rural communities.

Successful outcomes of this research could have far-reaching healthcare policy implications for rural CYP with medical complexity. A demonstrable change in health service use and acceptability of the model by families and health care personnel, could provide strong incentive for institutionalising this model of care, providing the opportunity for children to receive quality health care in a more equitable manner, closer to home.

## Conclusion

This study protocol is designed to conduct a robust evaluation of a rural paediatric care coordination intervention. The results of this study will provide an insight into the impact of this intervention on the child and family and the healthcare system within the Australian context. Moreover, the findings will provide evidence to inform practice and policy in national and international contexts.

## References

[1] Cohen E, Kuo DZ, Agrawal R, Berry JG, Bhagat SK, Simon TD, et al. Children with medical complexity: an emerging population for clinical and research initiatives. Pediatrics. 2011; 127(3): 529–38. DOI: 10.1542/peds.2010-0910 21339266 PMC3387912

[2] Peltz A, Hall M, Rubin DM, Mandl KD, Neff J, Brittan M, et al. Hospital Utilization Among Children With the Highest Annual Inpatient Cost. Pediatrics. 2016; 137(2): e20151829. DOI: 10.1542/peds.2015-1829 26783324 PMC9923538

[3] Breen C, Altman L, Ging J, Deverell M, Woolfenden S, Zurynski Y. Significant reductions in tertiary hospital encounters and less travel for families after implementation of Paediatric Care Coordination in Australia BMC Health Services Research. 2018; 18: 1–10. DOI: 10.1186/s12913-018-3553-4 PMC6171181 30285821

[4] Owens MK, editor Costs of uncoordinated care. The healthcare imperative: Lowering costs and improving outcomes: Workshop series summary; 2010. 21595114

[5] Satherley R-M, Green J, Sevdalis N, Newham JJ, Elsherbiny M, Forman J, et al. The Children and Young People’s Health Partnership Evelina London Model of Care: process evaluation protocol. BMJ Open. 2019; 9(8): e027302. DOI: 10.1136/bmjopen-2018-027302 PMC6731816 31481367

[6] Ziniel SI, Rosenberg HN, Bach AM, Singer SJ, Antonelli RC. Validation of a parent-reported experience measure of integrated care. Pediatrics. 2016; 138(6). DOI: 10.1542/peds.2016-0676 27940672

[7] Mumford V, Baysari MT, Kalinin D, Raban MZ, McCullagh C, Karnon J, et al. Measuring the financial and productivity burden of paediatric hospitalisation on the wider family network. Journal of paediatrics and child health. 2018; 54(9): 987–96. DOI: 10.1111/jpc.13923 29671913 PMC6635734

[8] Herdman M, Gudex C, Lloyd A, Janssen M, Kind P, Parkin D, et al. Development and preliminary testing of the new five-level version of EQ-5D (EQ-5D-5L). Quality of life research. 2011; 20(10): 1727–36. DOI: 10.1007/s11136-011-9903-x 21479777 PMC3220807

[9] Varni JW, Burwinkle TM, Seid M, Skarr D. The PedsQL™* 4.0 as a pediatric population health measure: feasibility, reliability, and validity. Ambulatory pediatrics. 2003; 3(6): 329–41. DOI: 10.1367/1539-4409(2003)003<0329:TPAAPP>2.0.CO;2 14616041

[10] Sweeney R, Chen G, Gold L, Mensah F, Wake M. Mapping PedsQLTM scores onto CHU9D utility scores: estimation, validation and a comparison of alternative instrument versions. Quality of Life Research. 2020; 29(3): 639–52. DOI: 10.1007/s11136-019-02357-9 31745690

[11] Clarke A, Friede T, Putz R, Ashdown J, Martin S, Blake A, et al. Warwick-Edinburgh Mental Well-being Scale (WEMWBS): validated for teenage school students in England and Scotland. A mixed methods assessment. BMC public health. 2011; 11(1): 1–9. DOI: 10.1186/1471-2458-11-487 21693055 PMC3141456

[12] Australian Bureau of Statistics. Census of Population and Housing: Socio-Economic Indexes for Areas (SEIFA) Australia 2016 [updated 27/3/18. Available from: https://www.abs.gov.au/ausstats/abs@.nsf/Lookup/by%20Subject/2033.0.55.001~2016~Main%20Features~IRSAD.

[13] Kontopantelis E, Doran T, Springate DA, Buchan I, Reeves D. Regression based quasi-experimental approach when randomisation is not an option: interrupted time series analysis. BMJ: British Medical Journal. 2015; 350: h2750. DOI: 10.1136/bmj.h2750 26058820 PMC4460815

[14] Damschroder L, Hall C, Gillon L, Reardon C, Kelley C, Sparks J, et al. The Consolidated Framework for Implementation Research (CFIR): progress to date, tools and resources, and plans for the future. Implementation Science. 2015; 10(1): A12. DOI: 10.1186/1748-5908-10-S1-A12

[15] Proctor E, Silmere H, Raghavan R, Hovmand P, Aarons G, Bunger A, et al. Outcomes for implementation research: conceptual distinctions, measurement challenges, and research agenda. Administration and policy in mental health and mental health services research. 2011; 38(2): 65–76. DOI: 10.1007/s10488-010-0319-7 20957426 PMC3068522

[16] Ponizovsky-Bergelson Y, Dayan Y, Wahle N, Roer-Strier D. A Qualitative Interview With Young Children: What Encourages or Inhibits Young Children’s Participation? International Journal of Qualitative Methods. 2019; 18: 1609406919840516. DOI: 10.1177/1609406919840516

[17] Finch TL, Girling M, May CR, Mair FS, Murray E, Treweek S, et al. Improving the normalization of complex interventions: part 2 – validation of the NoMAD instrument for assessing implementation work based on normalization process theory (NPT). BMC Medical Research Methodology. 2018; 18(1): 135. DOI: 10.1186/s12874-018-0591-x 30442094 PMC6238372

[18] Slaghuis SS, Strating MMH, Bal RA, Nieboer AP. A framework and a measurement instrument for sustainability of work practices in long-term care. BMC Health Services Research. 2011; 11(1): 314. DOI: 10.1186/1472-6963-11-314 22087884 PMC3234291

[19] Saldaña J. The coding manual for qualitative researchers: sage; 2021.

[20] Campbell JL, Quincy C, Osserman J, Pedersen OK. Coding in-depth semistructured interviews: Problems of unitization and intercoder reliability and agreement. Sociological methods & research. 2013; 42(3): 294–320. DOI: 10.1177/0049124113500475

[21] Tong A, Sainsbury P, Craig J. Consolidated criteria for reporting qualitative research (COREQ): a 32-item checklist for interviews and focus groups. International Journal for Quality in Health Care. 2007; 19(6): 349–57. DOI: 10.1093/intqhc/mzm042 17872937

[22] Turner SF, Cardinal LB, Burton RM. Research Design for Mixed Methods: A Triangulation-based Framework and Roadmap. Organizational Research Methods. 2015; 20(2): 243–67. DOI: 10.1177/1094428115610808

[23] QSR International Pty Ltd. NVivo qualitative data analysis software. 12 ed; 2018.

[24] Fereday J, Muir-Cochrane E. Demonstrating rigor using thematic analysis: A hybrid approach of inductive and deductive coding and theme development. International journal of qualitative methods. 2006; 5(1): 80–92. DOI: 10.1177/160940690600500107

[25] Damschroder LJ, Aron DC, Keith RE, Kirsh SR, Alexander JA, Lowery JC. Fostering implementation of health services research findings into practice: a consolidated framework for advancing implementation science. Implementation science: IS. 2009; 4(1): 50. DOI: 10.1186/1748-5908-4-50 19664226 PMC2736161

[26] McCartney M. Margaret McCartney: Breaking down the silo walls. BMJ. 2016; 354: i5199. DOI: 10.1136/bmj.i5199 27672074

[27] Perrin JM, Kuhlthau K, Walker DK, Stein RE, Newacheck PW, Gortmaker SL. Monitoring health care for children with chronic conditions in a managed care environment. Matern Child Health J. 1997; 1(1): 15–23. DOI: 10.1023/A:1026220201804 10728222

[28] Altman L, Breen C, Ging J, Burrett S, Hoffmann T, Dickins E, et al. “Dealing with the Hospital has Become too Difficult for Us to Do Alone” – Developing an Integrated Care Program for Children with Medical Complexity (CMC). Int J Integr Care. 2018; 18(3): 14. DOI: 10.5334/ijic.3953 PMC6144526 30245608

[29] Eastwood JG, Woolfenden S, Miller E, Shaw M, Garg P, Liu H, et al. Implementation, Mechanisms of Effect and Context of an Integrated Care Intervention for Vulnerable Families in Central Sydney Australia: A Research and Evaluation Protocol. Int J Integr Care. 2019; 19(3): 11. DOI: 10.5334/ijic.4217 PMC6659760 31367210

[30] NSW Ministry of Health. Strategic Framework for Integrating Care North Sydney NSW Ministry of Health; 2018. https://www.health.nsw.gov.au/integratedcare/Publications/strategic-framework-for-integrating-care.PDF.

[31] Australian Government. National Healthcare Agreement Australian Institute of Health and Welfare; 2021. https://meteor.aihw.gov.au/content/725844.

[32] Nicholson C, Jackson CL, Marley JE. Best-practice integrated health care governance – applying evidence to Australia’s health reform agenda. Med J Aust. 2014; 201(3 Suppl): S64–6. DOI: 10.5694/mja14.00310 25047885

